# Familial Aggregation of High Tumor Necrosis Factor Alpha Levels in Systemic Lupus Erythematosus

**DOI:** 10.1155/2013/267430

**Published:** 2013-09-25

**Authors:** Dorothy Mangale, Silvia N. Kariuki, Beverly S. Chrabot, Marissa Kumabe, Jennifer A. Kelly, John B. Harley, Judith A. James, Kathy L. Sivils, Timothy B. Niewold

**Affiliations:** ^1^Section of Rheumatology and Gwen Knapp Center for Lupus and Immunology Research, University of Chicago, Chicago, IL 60637, USA; ^2^Arthritis and Clinical Immunology Program, Oklahoma Medical Research Foundation, Oklahoma City, OK 73104, USA; ^3^Division of Rheumatology and The Center for Autoimmune Genomics & Etiology, Cincinnati Children's Hospital Medical Center, Cincinnati, OH 45229, USA; ^4^US Department of Veterans Affairs Medical Center, Cincinnati, OH 45229, USA; ^5^Division of Rheumatology and Department of Immunology, Mayo Clinic, 200 1st Street SW, Guggenheim building 3-42, Rochester, MN 55905, USA

## Abstract

Systemic lupus erythematosus (SLE) patients frequently have high circulating tumor necrosis factor alpha (TNF-**α**) levels. We explored circulating TNF-**α** levels in SLE families to determine whether high levels of TNF-**α** were clustered in a heritable pattern. We measured TNF-**α** in 242 SLE patients, 361 unaffected family members, 23 unaffected spouses of SLE patients, and 62 unrelated healthy controls. Familial correlations and relative recurrence risk rates for the high TNF-**α** trait were assessed. SLE-affected individuals had the highest TNF-**α** levels, and TNF-**α** was significantly higher in unaffected first degree relatives than healthy unrelated subjects (*P* = 0.0025). No Mendelian patterns were observed, but 28.4% of unaffected first degree relatives of SLE patients had high TNF-**α** levels, resulting in a first degree relative recurrence risk of 4.48 (*P* = 2.9 × 10^−5^). Interestingly, the median TNF-**α** value in spouses was similar to that of the first degree relatives. Concordance of the TNF-**α** trait (high versus low) in SLE patients and their spouses was strikingly high at 78.2%. These data support a role for TNF-**α** in SLE pathogenesis, and TNF-**α** levels may relate with heritable factors. The high degree of concordance in SLE patients and their spouses suggests that environmental factors may also play a role in the observed familial aggregation.

## 1. Introduction

Systemic lupus erythematosus (SLE) is a severe multisystem autoimmune disease which is caused by a combination of genetic and environmental factors [1]. Many lines of evidence underscore the importance of cytokines in SLE susceptibility. Circulating interferon alpha (IFN-*α*) levels are high in many SLE patients [2, 3]. One of the most direct lines of evidence suggesting that high IFN-*α* is a primary pathogenic factor is that some individuals treated with recombinant interferon alpha (IFN-*α*) for viral hepatitis develop de novo SLE, which typically resolves when IFN-*α* treatment is discontinued [4, 5]. Additionally, many of the genetic risk loci for SLE are in or near genes which play roles in cytokine pathways [6, 7]. A number of SLE-associated genes impact serum cytokine levels in SLE patients, providing further support for this idea [8, 9]. In the case of IFN-*α*, we have previously shown that high serum IFN-*α* is aggregated within SLE families, supporting the idea that high IFN-*α* is a heritable risk factor for SLE [10]. We have also demonstrated that high IFN-*α* is more common in family members of SLE patients who have a different non-SLE autoimmune disease, suggesting that high IFN-*α* may be a heritable factor predisposing to a number of autoimmune diseases [11]. Subsequent studies directed at defining the genetic architecture of the high IFN-*α* trait have implicated both established SLE and autoimmune disease risk genes [12–19], as well as novel genes which impact circulating IFN-*α* levels in SLE patients [20–24].

While much attention has been focused on IFN-*α* in SLE in recent years, many other cytokines will also play important roles in SLE pathogenesis. Serum tumor necrosis factor alpha (TNF-*α*) levels are elevated in many patients with SLE [13, 25, 26]. High levels of TNF-*α* have been correlated with increased clinical disease activity and the presence of anti-dsDNA antibodies [27]. High levels of TNF-*α* have been demonstrated in patients with lupus nephritis, and TNF-*α* is overexpressed in renal tissue in lupus nephritis [26, 28]. The role of TNF-*α* in murine models of SLE has been controversial. In some models TNF-*α* improved disease features [29], while in others TNF-*α* blockade has been beneficial [26]. Small scale clinical trials in human SLE suggest that short-term TNF-*α* blockade may have benefit in lupus nephritis, as well as transient benefit in SLE arthritis [26]. Significant side effects have been reported in a small group of SLE patients who have received long-term anti-TNF-*α* therapy [30], and there are no large-scale trials of TNF blockade in human SLE to date.

It is not clear whether high TNF-*α* predisposes to SLE or if the levels rise after the disease is established. Genetic studies have implicated a promoter polymorphism in the TNF-*α* gene in SLE susceptibility [31], although the TNF-*α* gene is within the HLA locus which is characterized by multiple association signals that are difficult to resolve due to high linkage disequilibrium in the region. It is also not clear that the TNF-*α* promoter polymorphism functionally confers a propensity for excess TNF-*α* mRNA or protein production [32]. In support of the idea that background genetic factors influence TNF-*α* levels, some non-HLA polymorphisms have been associated with differences in TNF-*α* in SLE patients [13, 24].

To explore this question further, we studied TNF-alpha levels in SLE families to determine whether high levels of TNF-alpha were heritable and aggregated in SLE families. We also examine the spouses of SLE patients to detect potential environmental contributions to familial tendencies. We also examine family TNF-*α* data in the context of IFN-*α* data in the same subjects from the same blood sample to detect potential relationships between these two cytokines in SLE patients and their families.

## 2. Methods

### 2.1. Patients and Samples

Serum and plasma samples were obtained from the Lupus Family Registry and Repository (LFRR) at the Oklahoma Medical Research Foundation and the Hospital for Special Surgery (HSS) Lupus Family Registry. There were no significant differences between the two cohorts in familial TNF-*α* or in the measures of familial clustering, and data from the two cohorts are presented in aggregate. A total of 206 samples from the HSS Lupus Family Registry were studied, including 106 SLE patients and 100 healthy family members. Clinical data are available for all samples in the registry, and serologic data are available for all of the SLE-affected individuals. A total of 397 samples from the LFRR were studied, including 136 SLE patients and 261 unaffected family members. Samples from 23 unaffected spouses were also available. Samples from 62 healthy unrelated controls were obtained from healthy blood donors. Demographic and clinical information for the SLE patients and healthy controls are shown in Table 1.

### 2.2. Measurement of TNF-*α* in Serum

TNF-*α* is measured using the Pierce Human Monoclonal TNF-*α* ELISA per manufacturer instructions. This ELISA has performed well in our hands to date with SLE samples [13, 25]. For categorical analyses, we used a cutoff for high TNF-*α* of two standard deviations above the mean of our nonautoimmune control population. Samples from families were not run together on the same plates or on the same days to prevent spurious correlations potentially related to a batch effect.

### 2.3. Reporter Cell Assay for IFN-*α*

The reporter cell assay for IFN-*α* has been described in detail elsewhere [10, 33]. Reporter cells were used to measure the ability of patient sera to cause IFN-*α*-induced gene expression. The reporter cells (WISH cells, ATCC #CCL-25) were cultured with 50% patient sera for 6 hours and then lysed. mRNA was purified from cell lysates, and cDNA was made from total cellular mRNA. cDNA was then quantified using real-time PCR using an Applied Biosystems 7900HT PCR machine with the SYBR Green fluorophore system. Forward and reverse primers for the genes *MX1*, *PKR*, and *IFIT1*, which are known to be highly and specifically induced by IFN-*α*, were used in the reaction [10]. *GAPDH* was amplified in the same samples to control for background gene expression. 

The amount of PCR product of the IFN-*α*-induced gene was normalized to the amount of product used for the housekeeping gene *GAPDH* in the same sample. The relative expression of each of the three tested IFN-induced genes was calculated as a fold increase compared to its expression in WISH cells cultured with media alone. Results from the IFN-*α* assay were standardized to a healthy multiancestral reference population as previously described, and a serum IFN-*α* activity score was calculated based upon the mean and SD of the reference population [10]. This assay has been highly informative when applied to SLE as well as other autoimmune disease populations [10, 34–37]. For categorical analyses, we used a cutoff for high IFN-*α* as of 2 standard deviations above the mean of the control group.

### 2.4. Statistical Analysis and Methods for Determining Familial Clustering and Heritability

Data from both TNF-*α* and IFN-*α* were nonnormally distributed. Median and interquartile range are used for graphical representation of quantitative data, and Mann-Whitney *U* test is used for comparison of groups. Correlation analyses were performed using the Spearman's rho rank order correlation. For categorical data analyses, we used the cutoffs noted above for each cytokine measurement to categorize subjects as high versus low. In the pedigree analyses, family members are first classified as SLE affected or unaffected. The unaffected individuals are then categorized by their closest relationship to an SLE-affected individual in the family. Each person in each registry is represented only once. Unaffected family members were classified by their most direct relationship to an SLE patient (for example, in an SLE family with multiple affected generations, sometimes a person could be both an SLE mother and an SLE grandmother—in this case the person is categorized as an SLE mother). Familial clustering is detected using the Fisher's exact test with categorical data, as outlined in [10]. Odds ratio (OR) for concordance in categorical IFN-*α* activity between SLE patients and their nuclear family members was calculated using a standard procedure, with input variables being the number of families with each of the following IFN-*α* activity patterns: patient high/family high, patient high/family low, patient low/family high, and patient low/family low. Relative recurrence risk ratios (*λ*) were calculated using a standard approach [38].

## 3. Results

### 3.1. Unaffected First Degree Relatives of SLE Patients had Higher TNF-*α* Than Unrelated Controls

**Figure 1:**
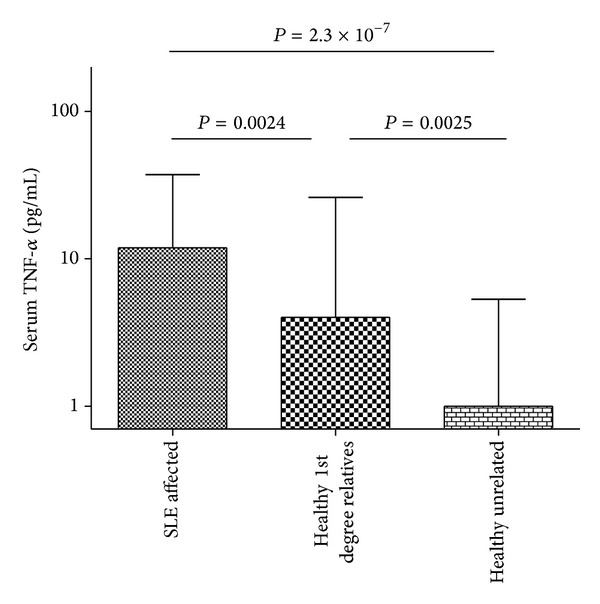
Serum TNF-*α* levels in SLE patients, first degree relatives, and unrelated controls. Bars show the median; error bars show the interquartile range, *P* values by Mann-Whitney *U* test.

Serum TNF-*α* was highest in the SLE patients, and TNF-*α* levels resembled those of previously published SLE cohorts [25]. Similar to previous work, we did not find any significant differences in serum TNF-*α* in the SLE patients by ancestral background or sex [25]. Patients had significantly higher levels of TNF-*α* than unaffected first degree relatives or unrelated controls (Figure 1, *P* = 0.0024 and *P* = 2.3 × 10^−7^, resp.). Interestingly, unaffected first degree relatives had significantly higher median serum TNF-*α* than unrelated controls (*P* = 0.0025). 

### 3.2. High TNF-*α* Was Strongly Aggregated in Nuclear SLE Families

No Mendelian patterns were observed in categorical analysis of TNF-*α* within the pedigrees. In this analysis, patients and relatives were categorized as having high versus low circulating TNF-*α* (see Section 2). 28.4% of unaffected first degree relatives of SLE patients had high TNF-*α* levels. We did observe strong familial aggregation of high TNF-*α* within SLE patients and their first degree relatives (*λ*_1st_ = 4.48, 2.9 × 10^−5^; see Table 2). Families with a high TNF-*α* SLE patient were likely to have a first degree relative with high TNF-*α*, and low TNF-*α* SLE patients were more likely to have low TNF-*α* relatives. As might be expected, the least common scenario was a patient with low TNF-*α* who had a first degree family member with high TNF-*α*. These analyses cannot be easily adjusted for factors such as disease activity or treatment in the SLE patient group because of course these factors are not applicable to the unaffected relatives. It is easy to think that an SLE patient might have had high TNF-*α* level which was decreased by aggressive immunosuppressive therapy, and this may result in a decreased correlation between the patient and their relatives. This of course would introduce a conservative bias into our study, biasing toward the null hypothesis. It is striking that we observe correlations between patients and family members despite some of these uncontrollable factors which may reduce the ability to detect familial correlations. We did not observe any significant increase in relative recurrence risk in second degree relatives (*P* = 0.24, data not shown).

### 3.3. Unaffected Spouses of SLE Patients Had TNF-*α* Levels, Which Were Highly Concordant with Their SLE Affected Spouse

**Figure 2:**
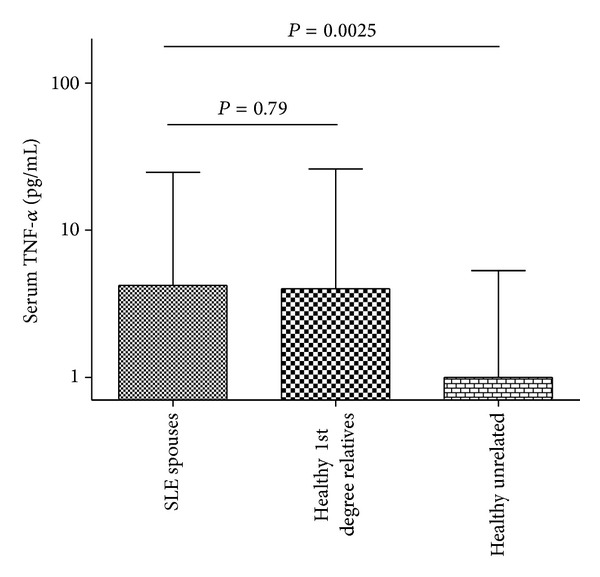
Serum TNF-*α* levels in first degree relatives of SLE patients, spouses of SLE patients, and unrelated controls. Bars show the median; error bars show the interquartile range, *P* values by Mann-Whitney *U* test.

Finding a correlation between closely related individuals in circulating TNF-*α* levels would suggest an inherited predisposition to high TNF-*α* within SLE families. Controlling environmental factors is difficult in human studies, but spouses provide an opportunity to study the unrelated individuals that share many environmental factors. We looked at circulating TNF-*α* levels in spouses of SLE patients and found that many spouses had high TNF-*α* levels, and as a group the median level resembled that of first degree relatives of SLE patients (Figure 2). Concordance of the TNF-*α* trait (high versus low) in SLE patients and their spouses was strikingly high at 78.2% in categorical analyses of TNF-*α* (OR = 3.60, *P* = 0.03, Table 3). 

### 3.4. IFN-*α* and TNF-*α* Were Correlated in SLE Patients, but Not in Unaffected Family Members

We observed evidence for a correlation between serum IFN-*α* and serum TNF-*α* levels in the SLE patients when comparing the measurements of these two cytokines from the same serum sample (Spearman's rho = 0.18, *P* = 0.0066). This is concordant with previous studies of concomitant TNF-*α* and IFN-*α* levels in SLE patients [25]. Interestingly, we did not observe the same correlation between these cytokines in first degree relatives of the SLE patients (Spearman's rho = 0.07, *P* = 0.25). This suggests that the factors underlying high IFN-*α* and high TNF-*α* within the SLE families are distinct, but converge upon the affected members of the families.

## 4. Discussion

In this study, we have demonstrated familial clustering of high TNF-*α* levels in SLE families. We have previously shown that high IFN-*α* is aggregated within SLE families [10] and that TNF-*α* and IFN-*α* are correlated to some degree in SLE patients [25], so perhaps it does not seem surprising that TNF-*α* is also clustered within SLE families. Important differences between the patterns of familial aggregation of these two cytokines that support the idea of the findings related to TNF-*α* reported in this study are not simply secondary to familial clustering of IFN-*α*. First, TNF-*α* levels in first degree relatives are not correlated with IFN-*α* levels, suggesting that different family members are contributing to the observed clustering of each of these cytokines in SLE families. In the SLE patients, a correlation is observed between these two cytokines, as has been observed in previous studies. This supports the idea that the factors predisposing to high levels of TNF-*α* and IFN-*α* within the SLE families are distinct and are generally present in different unaffected family members. These factors then presumably coalesce in the SLE affected members of the family. 

Secondly, the fact that spouses of SLE patients had high TNF-*α* levels which were frequently concordant between the patients and their spouses suggests an environmental factor leading to high TNF-*α* levels. This was not the case with IFN-*α* [10], as spouses did not have any significant increase in IFN-*α* above that of unrelated controls. Previous studies have documented a similar familial aggregation of IL-10 production from peripheral blood cells in SLE families, and in this study the familial correlation of IL-10 also extended to spouses of SLE patients [39]. Our study findings may be related to the findings in this IL-10 study, as high levels of TNF-*α* may induce a compensatory increase in IL-10, and both studies may be observing a similar phenomenon. We can only speculate about what environmental factor might induce increased TNF-*α* in SLE spouses and family members. Viral triggers of SLE have been proposed, and Epstein-Barr virus is a leading candidate in this regard [40]. Nearly everyone is infected with Epstein-Barr virus at some point in their lives, but there could be differences in quantitative exposures to Epstein-Barr virus within SLE families that could differ from healthy controls. These could relate with an increased propensity for subclinical reactivation in SLE patients, either related to the altered immunity produced by the disease itself or the immunosuppression given to treat the disease.

In previous studies of circulating IFN-*α* levels in SLE families, we have followed up familial clustering of the cytokine trait with evidence that a number of candidate genes are associated with high levels of IFN-*α* [41]. This has provided further support for heritability of IFN-*α*. With TNF-*α*, it is quite possible that we will identify candidate genes that are associated with this cytokine phenotype as well. An environmental effect on TNF-*α* levels within SLE families does not rule out genetic influences, and if genetic polymorphisms are associated with TNF-*α* levels, then the case for heritability would be strengthened. To date, we have observed one coding-change polymorphism in the ILT3 receptor which is associated with TNF-*α* levels in SLE patients [24]. Future work may establish additional genetic associations with the high TNF-*α* phenotype, and this would assist in defining differences in the molecular pathogenesis of SLE in different individuals affected by this heterogeneous condition.

## Figures and Tables

**Table 1:** 

	SLE patients	Unrelated controls
Age (yrs.)	40.8	45.6
Female gender	87.9	90.3
African-American	31.8	39.2
European-American	41.7	43.6
Hispanic-American	26.5	15.4
Malar rash	57.7	—
Discoid rash	8.3	—
Photosensitivity	43.5	—
Oral ulcer	31.0	—
Arthritis	75.0	—
Serositis	33.3	—
Renal d/o	42.9	—
Neuro d/o	13.7	—
Heme d/o	60.1	—
Immuno d/o	73.8	—
ANA	100.0	—
Anti-Ro	28.7	—
Anti-La	8.9	—
Anti-Sm	12.7	—
Anti-RNP	24.1	—
Anti-dsDNA	46.1	—

**Table 2:** 

TNF-*α* designation	No. of Instances	*P* value for familial aggregation	1st degree relative recurrence risk
High patient/high relative	63	2.9 × 10^−5^	4.48
High patient/low relative	86		
Low patient/high relative	46		
Low patient/low relative	166		

**Table 3:** 

TNF-*α* high versus low concordance	No. of couples	Odds ratio	*P* value
Concordant	18	3.60	0.03
Discordant	5		
